# Sex Differences and Commonalities in the Impact of a Palatable Meal on Thalamic and Insular Connectivity

**DOI:** 10.3390/nu12061627

**Published:** 2020-06-01

**Authors:** Lisa Kilpatrick, Teodora Pribic, Barbara Ciccantelli, Carolina Malagelada, Dan M. Livovsky, Anna Accarino, Deborah Pareto, Fernando Azpiroz, Emeran A. Mayer

**Affiliations:** 1 Division of Digestive Diseases, G Oppenheimer Center for Neurobiology of Stress and Resilience, Los Angeles, CA 90095, USA; lisa@lisakilpatrick.com (L.K.); emayer@ucla.edu (E.A.M.); 2 Digestive System Research Unit, University Hospital Vall d’Hebron, Centro de Investigación Biomédica en Red de Enfermedades Hepáticas y Digestivas (Ciberehd), Departament de Medicina, Universitat Autònoma de Barcelona, 08193 Bellaterra (Cerdanyola del Vallès), Spain; teodora.pribic@gmail.com (T.P.); bciccantelli@gmail.com (B.C.); cmalagelada@gmail.com (C.M.); aaccarino@telefonica.net (A.A.); 3 Digestive Diseases Institute, Shaare Zedek Medical Center, Hebrew University, 9103102 Jerusalem, Israel; danlivo@yahoo.com; 4 Radiology Department, University Hospital Vall d’Hebron, 08035 Barcelona, Spain; dpareto@idi-cat.org

**Keywords:** meal ingestion, postprandial sensations, hedonic response, brain imaging, sex differences, resting state

## Abstract

The neural mechanisms underlying subjective responses to meal ingestion remain incompletely understood. We previously showed in healthy men an increase in thalamocortical, and a decrease in insular-cortical connectivity in response to a palatable meal. As sex is increasingly recognized as an important biological variable, we aimed to evaluate sex differences and commonalities in the impact of a well-liked meal on thalamic and anterior insular connectivity in healthy individuals. Participants (20 women and 20 age-matched men) underwent resting-state magnetic resonance imaging (rsMRI) before and after ingesting a palatable meal. In general, the insula showed extensive postprandial reductions in connectivity with sensorimotor and prefrontal cortices, while the thalamus showed increases in connectivity with insular, frontal, and occipital cortices, in both women and men. However, reductions in insular connectivity were more prominent in men, and were related to changes in meal-related sensations (satiety and digestive well-being) in men only. In contrast, increases in thalamic connectivity were more prominent in women, and were related to changes in satiety and digestive well-being in women only. These results suggest that brain imaging may provide objective and sex-specific biomarkers of the subjective feelings associated with meal ingestion.

## 1. Introduction

Consumption of a meal induces a sequence of transient sensations before and during ingestion (e.g., anticipatory reward, gustation) as well as longer-lasting postprandial sensations. The postprandial experience involves homeostatic sensations (e.g., satiety) and hedonic sensations (e.g., digestive well-being), which are independently modulated by various conditioning factors [1]; for instance, more likable meals induce lower homeostatic sensations but stronger hedonic sensations [2]. The neural mechanisms underlying the postprandial experience remain incompletely understood [3]. While integration of neural and endocrine satiety signals occurs in the hypothalamus, the anterior insula plays a key role in the conscious sensation of palatability, hunger and satiation [4]. Indeed, alterations in these brain mechanisms have been reported in obesity [5]. We previously reported the results of a resting state functional magnetic resonance imaging (rsMRI) study examining the impact of a palatable meal on thalamic and insular brain networks in healthy men [6]. We found that thalamo-cortical connectivity increased and insular-cortical connectivity decreased with ingestion. Furthermore, in that study, meal-induced decreases in insular-anterior cingulate cortical (ACC) connectivity were related to more pronounced responses in self-reported satiety and digestive well-being.

Sex-related differences in the biological responses to food ingestion have been reported. Particularly, women experienced stronger postprandial sensations after a palatable meal, and were more susceptible to the influence of conditioning factors, such as the eating schedule [7,8,9]. Sex is increasingly recognized as an important biological variable influencing the generalizability of biomedical research [10]. In particular, sex differences have been observed in the structure and function of the insula, including differences in volume and tissue density [11], in the response to an autonomic challenge [12], pain-related alterations [13], structural connectivity [14], and functional connectivity [15]. Additionally, sex differences in gray matter volume and density have been shown in the thalamus [16]. Moreover, sex differences in brain responses to food-related cues and gustatory stimuli have been reported [16,17,18].

Based on these data, we hypothesized that the neural mechanisms underlying the postprandial experience exhibit sex differences. Thus, our aim is to examine sex differences and commonalities in the effects of a palatable meal on brain activity, specifically, on thalamic and anterior insular connectivity, and the relations of brain responses with homeostatic sensations (satiety) and hedonic sensations (digestive well-being).

## 2. Methods

### 2.1. Participants

Two groups of healthy, non-obese women and men (*n* = 20 each) without history of gastrointestinal symptoms participated in the study. The male participants represent a subset of the participants in a previously reported male-specific [6] and were chosen to match the women in terms of age. Since handedness influences brain organization, all participants were right-handed (as assessed by the Edinburgh Handedness Inventory) to include a homogeneous population for brain imaging studies [19]. Absence of current digestive symptoms was verified using a standard abdominal symptom questionnaire (no symptom ≥2 on a 0–10 scale); the reliability of this questionnaire has been validated by previous studies showing that it allows good discrimination between patients with functional digestive disorders and healthy subjects [20,21,22]. Individuals with psychological and eating disorders were excluded (as assessed by the following questionnaires: the Hospital Anxiety and Depression scale, Dutch Eating Behaviour Questionnaire, and the Physical Anhedonia Scale). Exclusion criteria were: history of anosmia, ageusia, current or recent dieting or any pattern of selective eating, such as vegetarianism, alcohol abuse, and the use of recreational drugs. For this pilot study no formal sample size calculations were performed. Participants were recruited by public advertisement in the university campus and received a monetary compensation for their participation. The study protocol, including recruitment and compensation procedures, was approved by the Institutional Review Board of the University Hospital Vall d’Hebron and was registered with Clinical Trials.gov as part of the study NCT02592239. All participants gave written informed consent.

### 2.2. Study Design and Procedure

To address our hypothesis we evaluated thalamic and insular connectivity by brain imaging and homeostatic and hedonic sensations by scales (outcome measures) in response to a palatable probe meal (intervention). Studies were conducted in the afternoon after a 5-h fast. Participants were instructed to refrain from strenuous physical activity on the day before the study and to have their usual breakfast after an overnight fast. Participants completed a structural MRI scan and two 20-min resting state MRI (rsMRI) scans (pre-1 and pre-2) immediately before ingestion of a probe meal, and a third 20-min rsMRI scan after ingestion (post meal); the two pre-meal rsMRI scans were acquired to examine the effect of time. Perception of subjective sensations was measured after the second fasting rsMRI scan (pre-meal), and immediately before and after the postprandial scan (0 min and 20 min post-meal, respectively). The probe meal was ingested while participants sat in an isolated room adjacent to the brain imaging room.

### 2.3. Intervention: Probe Meal

To evaluate the brain activity underlying a positive postprandial experience, a probe meal (comfort-type meal) was specifically designed by a series of previous studies [23]; the characteristics of the meal (amount, composition, and palatability) were tailored to induce a consistent homeostatic response (satiation) with a pleasurable hedonic dimension (digestive well-being). The probe meal (425 kcal; 47 g carbohydrates, 17 g lipids, 18 g proteins) consisted of 200 mL orange juice and a warm sandwich (58 g white bread with 12 g butter, 38 g ham and 38 g cheese) freshly cooked on a hot plate (Sandwich Maxi 20, Fagor, Olite, Spain) for 3 min and administered at a standard temperature (allowed to cool down covered by a napkin for 3 min).

### 2.4. Outcome Measures

#### 2.4.1. Assessment of Subjective Responses

Ten centimeter scales graded from −5 to +5 were used to measure hunger/satiety (extremely hungry to completely sated), digestive well-being (extremely unpleasant sensation/dissatisfaction to extremely pleasant sensation/satisfaction), and palatability (very bad/disagreeable to very good/delicious). Subjects received standard instructions on how to complete the scales: The hunger/satiety and digestive well-being scales were scored immediately before, at the end and 20 min after meal ingestion (score the intensity of each sensation you feel now); the palatability scale (how did you find the meal) was scored only once immediately after ingestion. These scales have been shown to detect differences in postprandial sensations under various conditioning factors [1,2,9,24].

#### 2.4.2. Neuroimaging

fMRI acquisition. Images were acquired on a 3.0 T whole-body MR scanner with a 12-channel phased-array head coil and a whole-body transmit coil (Trio, Siemens, Germany). The imaging protocol parameters were based on the ADNI initiative.The structural scan involved an axial 3D T1-weighted gradient-echo (MPRAGE) sequence (TR = 2200 ms, TE = 3.26 ms, voxel size = 1.0 × 1.0 × 1.0 mm^3^). Three 20-min rsMRI scans (pre-1, pre-2 and post ingestion) were completed, in which participants rested with eyes closed (TR = 2000 ms, TE = 28 ms, voxel size = 3.4 × 3.4 × 4.0 mm^3^).

fMRI preprocessing. Imaging data were pre-processed using SPM12 software (Welcome Department of Cognitive Neurology, London, UK). The first two volumes were discarded to allow for stabilization of the magnetic field. Data were slice-time and motion corrected, spatially normalized to the Montreal Neurological Institute standard template using their structural image, and smoothed with an 8-mm Gaussian kernel. In addition, white matter/cerebrospinal fluid signals were regressed out of the time series data, which were then bandpass filtered (0.01–0.10 Hz).

### 2.5. Statistical Analysis

#### 2.5.1. Perception Measurements

Mean values (±SE) of the parameters measured were calculated in each group of subjects. Normality of data distribution was evaluated by the Kolmogorov-Smirnov test. Comparisons of parametric normally distributed data were made by Student’s *t* test, paired tests for intragroup comparisons and unpaired tests for intergroup comparisons; otherwise, the Wicoxon signed rank test was used for paired data within groups, and the Mann-Whitney U test for unpaired data between groups. Differences were considered significant at a *p* value < 0.05.

#### 2.5.2. Brain Connectivity

Seed-based correlation has been used in the analysis of rsMRI data, as previously described in detail [25]. In brief, this method provides direct and easily interpreted information regarding connectivity for pre-defined regions of interest. The left and right short insula gyri and left and right thalamus, based on the Destrieux atlas [26], were utilized as regions of interest (ROIs) in the present study. After regressing out the motion parameters and bandpass filtering (0.01–0.08 Hz) to reduce noise, the mean time course was extracted from each of the ROIs, and correlated with the time courses of all other grey matter voxels. The resulting correlation maps were normalized using Fisher’s r-to-z transformation [27].

Partial least squares (PLS) analysis was applied to the normalized correlation maps to examine sex-related and meal-ingestion effects on anterior insula and thalamic connectivity, using freely available code [28]. PLS is a multivariate statistical technique that is used to find the relationship between two blocks of variables, with numerous advantages; for example, unlike SPM, PLS does not require a prior specification of the contrasts of interest, and PLS is more sensitive than traditional univariate SPM-based analyses [28,29].

Four seed-PLS analyses were conducted, one for each of the four ROIs (left and right anterior insula; left and right thalamus). In each analysis, sex was a group factor and there were three meal-ingestion conditions (pre-1, pre-2, and post-meal); the normalized correlation maps were provided as input. As a result, each seed-PLS analysis produced a set of spatial maps with associated weightings for each combination of sex and condition (i.e., latent variables). Significance of each latent variable was assessed using permutation testing (500 permutations), and voxel reliability was assessed using bootstrap estimation (500 samples) [28]. Clusters with a peak voxel bootstrap standard error ratio (BSR) exceeding ±3.3 (*p* < 0.001) and an extent of at least 150 voxels were considered as reliable.

#### 2.5.3. Correlation of Brain Connectivity and Sensations

Reliable clusters from the seed-PLS analyses were further analyzed in focused behavioral-PLS analyses, which evaluated the relationships between connectivity changes and changes in perceptual ratings within each sex. As the seed-PLS analyses showed no significant differences between the first and second pre-meal ingestion scan, connectivity difference maps were calculated as the post-meal ingestion connectivity map minus the second pre-meal ingestion connectivity map (post minus pre-2). The ingestion-related changes in satiety and digestive well-being ratings (Δ satiety and Δ well-being) were calculated as the post-meal ingestion rating minus the pre-meal ingestion rating. For each sex, four behavioral-PLS analyses were conducted, one for each of the four ROIs; the connectivity difference maps and both Δ satiety and Δ well-being were provided as input. As a result, each behavioral-PLS analysis produced a set of spatial maps with associated weightings for Δ satiety and Δ well-being (i.e., latent variables). Significance of each latent variable and voxel reliability were assessed in the same manner as in the seed-PLS analyses.

## 3. Results

### 3.1. Demographics

Age range was similar in women (20–43 years) and men (20–40 years). Body mass index was also similar in women (range 19–29 Kg/m^2^) and men (20–29 Kg/m^2^); one participant in each group was overweight and the rest were within the normal range. Participants were university students or had a university degree (middle-upper class) and all completed the study and were included in the final analysis.

### 3.2. Meal-Related Sensations

**Figure 1:**
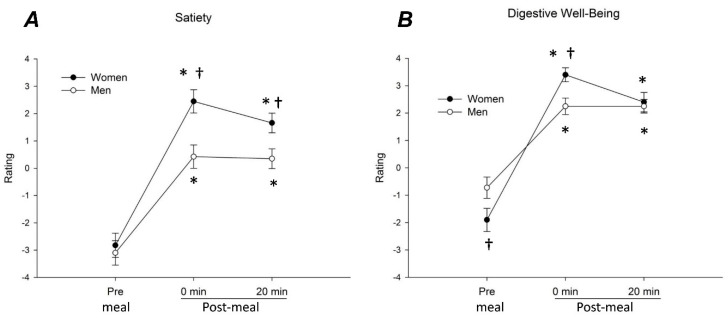
Sensory responses to meal ingestion. Satiety (**A**) and digestive well-being (**B**) ratings (mean values and SE) before (Pre) and at 0 and 20 min after meal ingestion. Note more prominent homeostatic response (satiety) and early hedonic response (digestive well-being) in women than in men.* *p* < 0.05 vs. Pre (significant ingestion-related change); † *p* < 0.01 vs. men (significant sex difference within condition).

Both men and women found the probe meal equally likeable (palatability score 3.6 ± 0.2 in women and 3.9 ± 0.2 in men; *p* = 0.210). Meal ingestion increased satiety and digestive well-being ratings in men and women (*p* < 0.001 for all) and these effects persisted over the 20-min postprandial period (*p* < 0.001 for all) (Figure 1). In women, satiety ratings at 0 and 20 min after meal ingestion were higher than in men (*p* = 0.003 for both) (Figure 1A). Sex differences were also observed in relation to digestive well-being: before the meal, well-being ratings were higher in men than in women (*p* = 0.048), but ratings immediately after the meal were higher in women than in men (*p* = 0.006); hence, the increase in well-being (Δ well-being) was larger in women than in men (Figure 1B). At 20 min after ingestion well-being rates were similar in women and men (Figure 1B)

### 3.3. Brain Imaging

No significant differences between the two pre-meal ingestion scans were detected both in women and men, indicating the lack of time effects before the intervention (meal ingestion); in contrast, marked and consistent changes were detected after meal ingestion.

#### 3.3.1. Anterior Insular Connectivity

**Figure 2:**
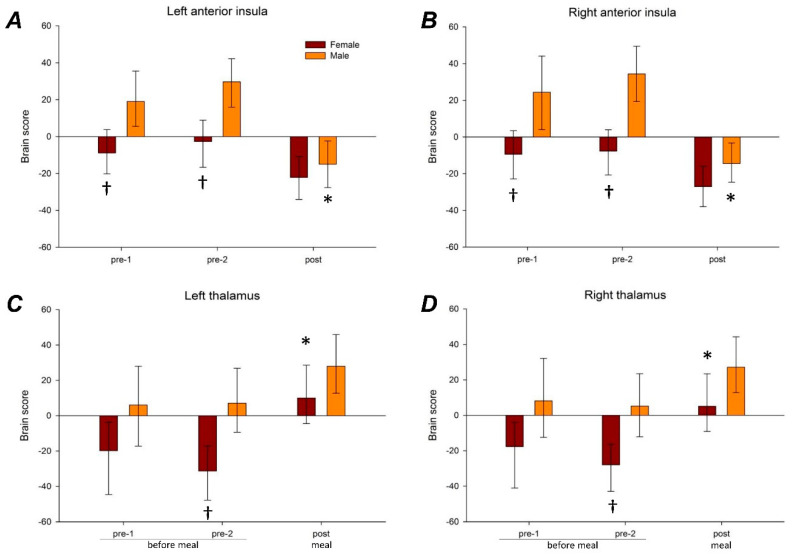
Brain responses to meal ingestion. Brain connectivity summary scores (mean and confidence interval) in the two sequential scans before the meal (pre-1 and pre-2) and in the scan after the meal (post) are plotted according to sex and condition for each region of interest: (**A**) Left anterior insula; (**B**) right anterior insula; (**C**) left thalamus; and (**D**) right thalamus. The brain connectivity summary scores reflect the weightings (analogous to factor scores in a factor analysis) determined by the partial least squares analysis. The brain regions reliably contributing to these weightings are shown in Figure S1. * *p* < 0.05 vs. Pre (significant ingestion-related change); † *p* < 0.05 vs. men (significant sex difference within condition).

Significant connectivity patterns (latent variables) were detected for right anterior insular (accounting for 72.3% of the cross-block variance; *p* < 0.001) and for left anterior insula (accounting for 66.1% of the cross-block variance; *p* < 0.001). Before the meal, i.e., at baseline, significant sex differences in connectivity for the right and left anterior insula were observed (Figure 2A,B): specifically, connectivity between the anterior insula and sensorimotor and prefrontal cortices was greater in men than in women during both pre-1 scan and pre-2 scan (Table 1 and Figure S1A,B). In men meal ingestion significantly reduced connectivity of the left and right anterior insula with these regions; in women the change was not significant and tended to be smaller (although not significantly) than in men, so that sex differences in connectivity observed at baseline were no longer detectable after meal ingestion (Figure 2A,B).

#### 3.3.2. Thalamic Connectivity

Significant connectivity patterns (latent variables) were detected for right thalamus (accounting for 62.4% of the cross-block variance; *p* = 0.002) and for left thalamus (accounting for 66.2% of the cross-block variance; *p* = 0.002). During baseline, sex differences in connectivity for the right and left thalamus were observed, reaching statistical significance at the pre-2 scan (Figure 2C,D); specifically, connectivity between thalamus and insular, frontal, and occipital cortices were greater in men than women (Table 1 and Figure S1C,D). Meal ingestion had opposite effects on the anterior insula and thalamus connectivities. In contrast to the decrease in the anterior insula connectivity (described above), meal ingestion increased the connectivity between the thalamus and the regions shown in Table 1 and Figure S1C,D. In women meal ingestion significantly increased connectivity of the right and left thalamus with these regions; in men the changes were not significant and tended to be smaller (although not significantly), so that the sex differences in connectivity observed at the pre-2 scan were no longer significant after meal ingestion.

#### 3.3.3. Correlations between Brain Activity and Sensations

The correlations between sensations and brain activity were different in women and men.

In women, the increase in satiety and well-being induced by meal ingestion was significantly and directly correlated with meal-induced changes in connectivity of the right and left thalamus (Table 2); hence, greater satiety and well-being were associated with greater increases in the connectivity of the thalamus (both right and left) with temporal and insular regions (mid and posterior) (Figure S2A). In contrast, no significant correlations between meal-induced satiety and well-being with changes in right and left anterior insular connectivity were observed.

In men, no significant correlations between meal-induced effects on satiety and well-being with the effects on thalamic connectivity (both right and left) were detected. However, the increase in satiety and well-being exhibited significant inverse correlations with changes in connectivity of the left anterior insula and direct correlations with right anterior insula, (Table 2); thus, greater satiety and well-being were associated with a larger reduction in the connectivity of the left anterior insula with the left temporoparietal junction (TPJ) and greater increase in the connectivity of the right anterior insula with frontal regions (Figure S2B).

## 4. Discussion

Our study demonstrates that ingestion of a palatable meal induces a response in brain activity and subjective meal-related sensations common to women and men, but with specific sex differences.

The probe meal used in the present study was specifically designed to induce a consistent and pleasurable postprandial experience [23] and, as expected it was found palatable and induced positive subjective feelings of satiety and digestive well-being. In accordance with previous studies, these sensations were associated with decreases in anterior insular connectivity and increases in thalamic connectivity [6]. The present study shows that this pattern is common to women and men; however, the study identified sex differences regarding subjective ratings of meal effects, brain connectivity, and the relations between them.

As in previous studies [7,8], compared to men, women experienced more satiety and satisfaction after the palatable meal. It has been shown that both appetite [24] and the initial gustatory experience [2] may influence postprandial sensations; however, in the present as well as in previous studies sex-related differences in postprandial sensations were not related to these factors, because pre-meal hunger scores and palatability ratings of the meal were similar in both groups. Homeostatic and hedonic sensations after a meal depend on the characteristics of the meal itself and the receptivity of the eater. Conceivably sex differences depend on constitutive factors, but the potential influence of inducible factors susceptible to conditioning by previous experience and education, cannot be ruled out. Indeed, we acknowledge that we cannot ascertain whether the differences observed are related to sex (constitutive, i.e., biological) or gender (inducible, i.e., role).

Notwithstanding the greater subjective responses observed in women, anterior insular connectivity changes in response to meal ingestion (decrease) were more prominent in men. Previous studies showed a more prominent anterior insula response to chocolate satiation [30], and greater change from hunger to satiety in the insula’s response to a variety of tastes in men compared with women [16]. These data suggest that the insula is more reactive to food ingestion in men; however in the present study, anterior insular connectivity in the postprandial state was similar in men and women, and the differences were related to the fasting state, suggesting that anterior insular connectivity may be more sensitive to fasting conditions in men compared to women.

Our study further shows that sex differences are involved in the way that specific changes in brain connectivity relate to the subjective experience of the meal effects. Hence, the reductions in anterior insular connectivity were related to changes in perception (satiety and digestive well-being) in men only. Indeed, in men, but in not women, greater Δ satiety and Δ well-being were associated with changes in anterior insula connectivity, specifically, with the TPJ and precentral and prefrontal cortices. The anterior insular cortex is a key node in the brain’s salience network, and is consistently activated in response to salient, homeostatically relevant stimuli associated with conscious feelings such as hunger and satiation [31]. The TPJ is a key node in the ventral attention network, responds to salient, behaviorally relevant stimuli, and plays a role in redirecting attention [32,33]. Greater Δ satiety and Δ well-being in response to meal ingestion were associated with a greater reduction in connectivity (i.e., less coordination) between these salience-related nodes. In contrast, although right anterior connectivity with inferior frontal and midfrontal cortices generally decreased with meal ingestion, larger reductions were associated with smaller Δ satiety and Δ well-being. As the anterior insula, inferior frontal, and midfrontal cortices are involved in perceptual updating [34], reduced coordination among these regions may disrupt the updating of ingestion-related status.

The thalamus showed a pattern of results opposite to those of the anterior insula; thalamic connectivity increased in response to meal ingestion and the changes were more prominent in women than in men. As with the anterior insula, thalamic connectivity in the postprandial state was very similar in men and women, while sex differences were observed before meal ingestion. Thus, thalamic connectivity may also be differentially sensitive to fasting conditions in women and men, specifically, with TPJ, precentral, and prefrontal cortices. Additionally, in women, but not in men, greater Δ satiety and Δ well-being were associated with increases in thalamic connectivity, specifically, with the mid and posterior insula and temporal cortices. The thalamus conveys sensory information from the body to the insula, with an extensive relationship between the ventroposterior medial nucleus of the thalamus and the anterior insula. The dorsal mid-insular cortex has been implicated as a region of convergence between gustatory and interoceptive information [35,36,37,38,39]. Thus, greater Δ satiety and Δ well-being in response to meal ingestion may depend on increased coordination between the thalamus and mid-insula.

We wish to acknowledge that the generalizability of our findings is limited by the small sample size and will require repetition in a larger sample. Our observations are limited to the responses of specific brain regions to a pleasant meal. Based on the literature, brain regions other than insula and thalamus are involved in the brain response to a meal, including the basal ganglia and the hypothalamus, which may also contribute to sex-related differences. Furthermore, sex-differences in the brain responses to non-palatable or aversive meals remain to be explored. As described in another article of this special issue [1], previous studies proved the validity of the scales to detect differences in the sensory responses to meals under different conditions, but a potential bias related to this methodology may be involved.

## 5. Conclusions and Implications

The results of the present study have general implications for food-related neuroimaging research in healthy individuals, as well as in patient populations (e.g., obesity, dyspepsia) [18]. Our findings suggest that perceptions of postprandial satiety and digestive well-being are a reflection of specific connectivity changes of two major integrative hubs of the brain, the anterior insula and thalamus, which receive sensory information (including gustatory and homeostatic information) and project to a wide range of cortical regions, in a sex-specific manner. Thus, although brain imaging may provide objective biomarkers of the subjective effects of meal ingestion, these biomarkers appear to be sex-specific. Future ingestion-related studies in patient populations with altered postprandial sensations, such as in functional dyspepsia, should further examine the impact of sex. Furthermore, the postprandial experience, i.e., the long lasting sensations after ingestion, may be key to food preferences, and understanding sex differences in this context may be important in the development of educational programs to modify eating habits both in patients with specific requirements and in the general population.

## Figures and Tables

**Table 1:** Regions of interest (ROI) connectivity affected by meal ingestion.

Seed Region	X	Y	Z	BSR	Num.	Pre-1 r_z_		Pre-2 r_z_		Post r_z_	
	mm	mm	mm		voxels	F	M	F	M	F	M
**Left anterior insula**											
**L precentral, postcentral**	−60	8	28	6.75	8808	0.20	0.39	0.25	0.45	0.15	0.17
R insula	40	−32	18	6.72	6054	0.24	0.48	0.32	0.56	0.18	0.30
Bilat precuneus	4	−54	10	6.16	4470	0.06	0.27	0.12	0.33	0.01	0.07
Bilat medial superior frontal	2	56	16	5.63	2180	0.19	0.35	0.23	0.40	0.10	0.14
R precentral, postcentral	32	−20	50	5.62	1223	0.10	0.24	0.14	0.26	0.01	0.00
L SMA	−12	0	70	5.19	420	0.21	0.31	0.16	0.39	0.16	0.19
L superior frontal	−16	28	46	5.18	368	0.03	0.17	0.08	0.18	−0.08	0.14
Bilat paracentral lobule	−4	−24	68	4.74	549	0.16	0.25	0.22	0.37	0.08	0.12
**Right anterior insula**											
L insula, Bilat postcentral, precentral, paracentral lobule, inferior frontal, SMA	−36	−26	22	7.46	30687	0.16	0.43	0.23	0.47	0.08	0.28
Bilat lingual	12	−44	−8	6.54	4286	0.02	0.25	0.07	0.30	−0.08	0.10
Bilat medial superior frontal	2	66	18	5.88	4002	0.03	0.28	0.07	0.31	0.00	0.06
R inferior frontal	26	24	−2	5.51	910	0.19	0.30	0.28	0.43	0.20	0.16
L precuneus	−16	−76	38	4.52	150	0.28	0.40	0.27	0.46	0.24	0.30
R calcarine	8	−98	−8	4.48	193	−0.08	0.15	−0.17	0.17	−0.10	0.05
**Left thalamus**											
R superior parietal, angular	28	−68	54	6.44	1463	0.03	0.21	0.01	0.21	0.17	0.32
R fusiform	38	−40	−18	6.40	1366	−0.08	0.05	−0.14	0.06	0.05	0.12
R medial superior frontal	14	62	30	6.39	637	0.06	0.30	−0.01	0.24	0.25	0.37
R mid frontal	40	52	−4	6.35	2734	−0.02	0.12	−0.09	0.05	0.19	0.25
L fusiform	−46	−54	−14	6.34	1165	−0.07	0.12	−0.09	0.13	0.12	0.25
R putamen, insula	36	0	−2	6.31	2286	0.01	0.18	−0.12	0.14	0.08	0.28
L putamen, insula	−38	−6	−2	5.96	2890	0.03	0.20	0.00	0.16	0.19	0.41
L inferior frontal	−44	28	0	5.73	910	0.05	0.17	−0.07	0.06	0.13	0.34
L mid occipital	−32	−88	16	5.54	1739	−0.02	0.10	−0.15	0.08	0.14	0.24
R superior frontal	16	38	50	5.52	582	0.08	0.17	−0.06	0.11	0.25	0.29
L superior frontal	−18	32	56	5.22	154	0.10	0.21	0.03	0.25	0.21	0.38
R medial OFC	6	56	−4	5.11	480	0.14	0.27	0.06	0.24	0.23	0.35
L calcarine	−8	−58	8	5.05	772	0.03	0.17	−0.01	0.21	0.23	0.33
L inferior parietal	−28	−46	40	4.38	150	0.00	0.12	−0.08	0.06	0.11	0.17
**Right thalamus**											
R putamen, R insula	34	2	−2	6.75	4820	0.09	0.22	−0.07	0.15	0.17	0.33
R fusiform	40	−50	−18	5.78	861	−0.12	−0.04	−0.15	0.02	0.01	0.20
R superior parietal	28	−68	54	5.58	494	0.10	0.21	0.06	0.22	0.18	0.34
**L anterior insula, inferior frontal gyrus**	−46	16	−2	5.51	917	0.01	0.16	−0.01	0.21	0.23	0.26
**R mid occipital gyrus**	36	−84	24	5.38	212	−0.05	0.11	−0.15	0.07	0.08	0.18
**L fusiform gyrus**	−48	−54	−14	5.27	261	−0.01	0.16	−0.06	0.16	0.14	0.21
**L posterior insula**	−36	−6	0	5.20	1498	0.02	0.22	0.01	0.14	0.13	0.39
**R mid frontal gyrus**	22	44	28	5.07	609	0.06	0.17	0.00	0.15	0.13	0.28
**Bilat SMA**	2	26	58	5.00	307	0.03	0.30	0.07	0.33	0.24	0.31
**R medial OFC**	6	54	−2	4.87	280	0.13	0.27	0.07	0.25	0.22	0.30
**L angular gyrus**	−40	−54	24	4.84	368	−0.05	0.11	−0.11	0.04	0.11	0.14
**L mid occipital**	−28	−88	16	4.55	296	−0.06	0.07	−0.12	0.05	0.06	0.20
**L cerebellum**	−18	−74	−28	4.41	435	0.19	0.35	0.19	0.30	0.30	0.42

Only statistically significant values are shown; (*p* < 0.001 for all). The seed region is shown in italics. The z-transformed correlation coefficient (r_z_), reflecting the strength of connectivity of the peak voxel with the seed region for each condition/sex, is reported to aid in the interpretation of the partial least squares analysis. Bilat, bilateral; BSR, bootstrap ratio; F, female; L, left; M, male; OFC, orbitofrontal cortex; R; right; ROI, region of interest; SMA, supplemental motor area.

**Table 2:** Ingestion-related ROI connectivity changes correlated with change in perceptual ratings.

Region	X mm	Y mm	Z mm	BSR	Size Voxels	Well-Being r	Satiety r
**FEMALES**							
**Left thalamus**							
R mid-temporal	64	−46	−4	7.25	391	0.74	0.53
R fusiform gyrus	40	−42	−22	5.45	217	0.69	0.69
L pINS	−32	−32	16	4.70	254	0.61	0.38
**Right thalamus**							
L pINS	−36	−26	10	6.45	291	0.70	0.33
R midINS	40	−4	−14	6.15	177	0.60	0.44
R fusiform gyrus	28	−40	−12	4.96	171	0.50	0.54
**MALES**							
**Left anterior insula**							
**L TPJ**	−44	−44	22	5.38	191	−0.72	−0.56
**Right anterior insula**							
**R inferior frontal**	42	16	22	5.43	288	0.69	0.40
**L precentral**	−32	−6	50	5.04	427	0.65	0.46
**R midfrontal**	36	10	46	−4.87	288	0.57	0.53

Only statistically significant correlations are showed (*p* < 0.001 for all). The seed region is shown in italics. The z-transformed correlation coefficient (r_z_), reflecting the correlation between perceptual ratings and connectivity of the peak voxel with the seed region for each condition/sex, is reported to aid in the interpretation of the partial least squares analysis. BSR, bootstrap ratio; midINS, mid-insula; pINS, posterior insula; ROI, region of interest; TPJ, temporoparietal junction.

## Supplementary Materials

The following are available online at https://www.mdpi.com/2072-6643/12/6/1627/s1, Figure S1: Ingestion-related brain connectivity changes; Figure S2: Relation between brain responses and perception induced by meal ingestion.
