# Supplementary material for: Sex Differences and Commonalities in the Impact of a Palatable Meal on Thalamic and Insular Connectivity
Source: Nutrients. 2020 Jun 1;12(6):1627. doi: 10.3390/nu12061627 (PMC7352462; doi:10.3390/nu12061627)
Supplement: Supplementary file 1 [file nutrients-12-01627-s001.pdf]

<u>Figure S1</u>: Ingestion-related brain connectivity changes.

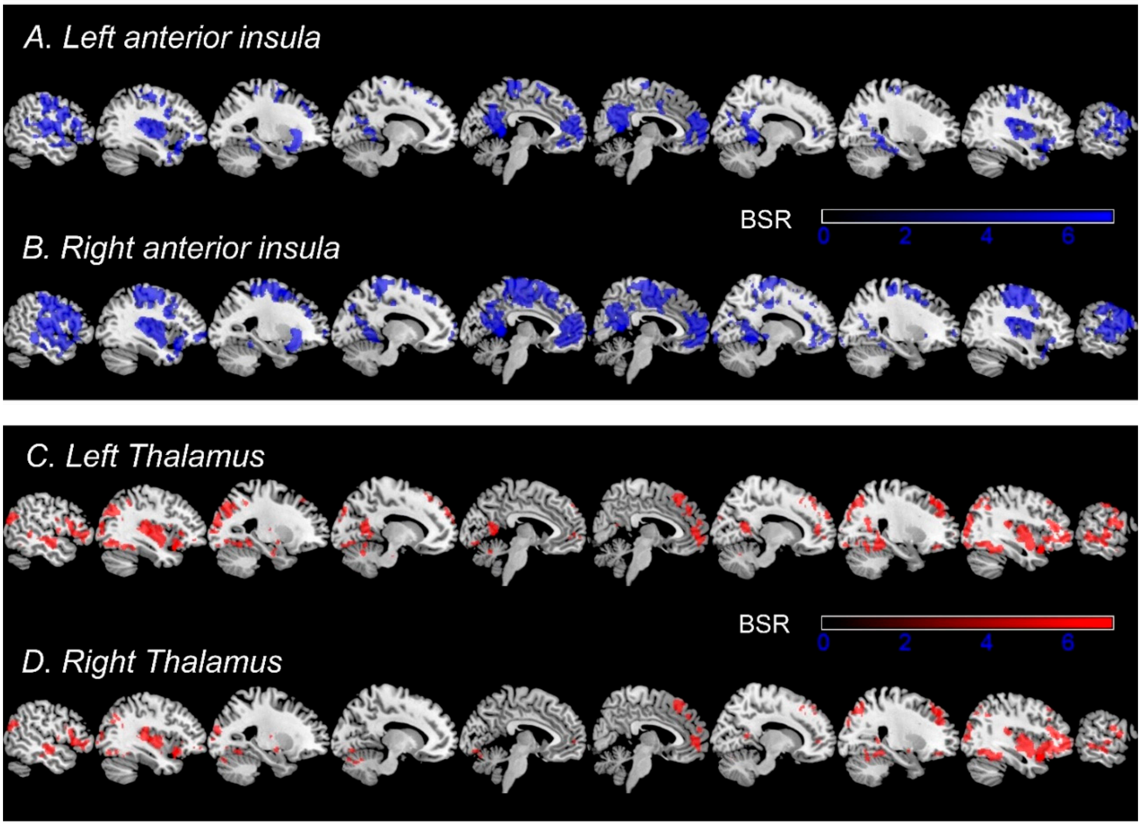

Ten sagittal planes of the brain (from right to left) depict regions showing significant ingestion-related connectivity changes with the left anterior insula (A), right anterior insula (B), left thalamus (C), and right thalamus (D) (i.e., the four regions of interest). Blue indicates decreased connectivity with meal ingestion (which tended to be larger in men than in women) and red increased connectivity with meal ingestion (which tended to be larger in men than in women); pooled data for women and men. BSR, bootstrap standard error ratio.

Figure S2: Relation between brain responses and perception induced by meal ingestion.

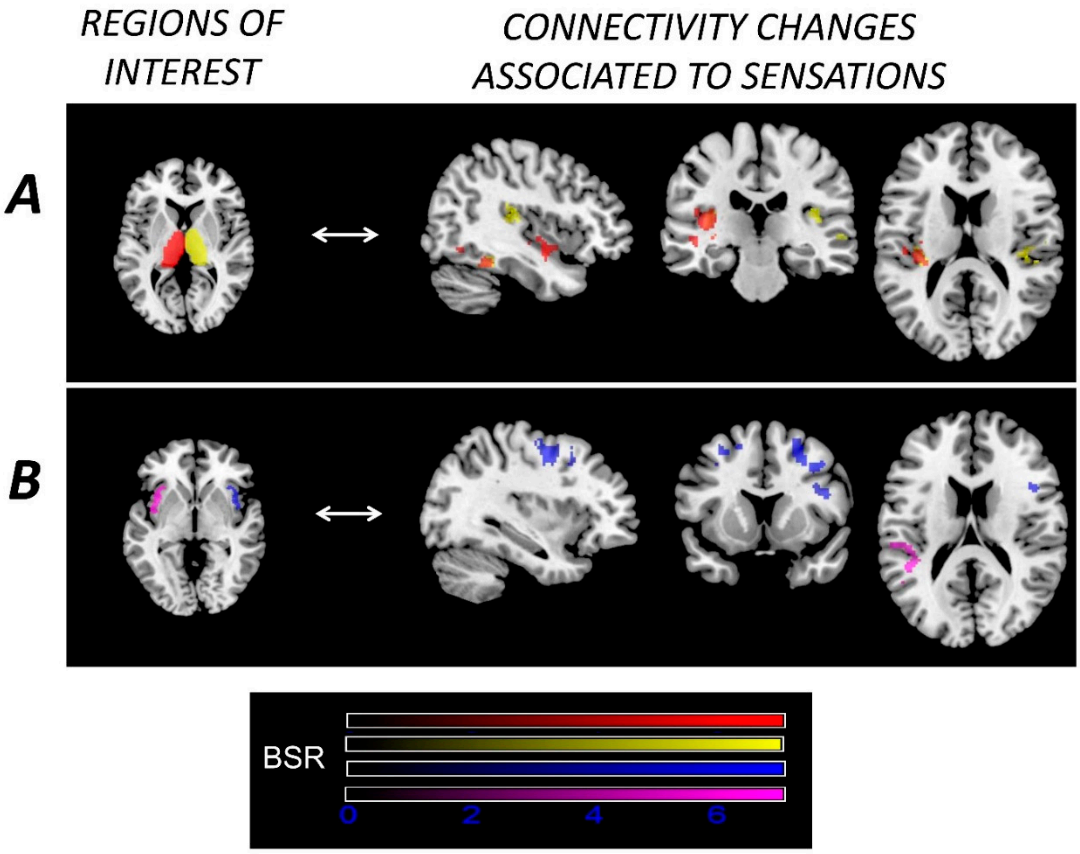

Connectivity changes related to increased satiety (Δ satiety) and digestive well-being (Δ well-being) in women (A) and men (B). The regions of interest are shown color-coded on the left, and the respective connectivity changes significantly associated with Δ satiety and Δ well-being are shown on the right using the same color-coding. (A) In women, thalamic, but not anterior insular, connectivity changes were significantly associated with both Δ satiety and Δ well-being. Overlap in left thalamic (red) and right thalamic (yellow) connectivity changes results in an orange hue. (B) In men, anterior insular, but not thalamic, connectivity changes were significantly associated with both Δ satiety and Δ well-being. There was no overlap in left anterior insula (violet) and right anterior insula (blue) connectivity changes associated with Δ satiety and Δ well-being. BSR, bootstrap standard error ratio.
